# Protocol for a systematic review of the efficacy of fat grafting and platelet-rich plasma for wound healing

**DOI:** 10.1186/s13643-017-0505-8

**Published:** 2017-06-06

**Authors:** Oliver J. Smith, Muholan Kanapathy, Ankur Khajuria, Max Prokopenko, Nadine Hachach-Haram, Haroon Mann, Ash Mosahebi

**Affiliations:** 1 Royal Free Hospital Wound Healing Group, Department of Plastic and Reconstructive Surgery, Royal Free Hospital, London, UK; 2 Division of Surgery and Interventional Science, University College London, London, UK; 3 Academic Surgery Foundation Programme, St Mary’s Hospital, Imperial College London, London, UK; 4 Department of Trauma and Orthopaedics, Royal Free Hospital, London, UK

**Keywords:** Fat grafting, Lipofilling, Platelet rich plasma, Wound healing, Systematic review

## Abstract

**Background:**

The use of fat grafting as a reconstructive surgical option is becoming much more common. Adipose-derived stem cells found in fat grafts are believed to facilitate wound healing via differentiation into fibroblasts and keratinocytes and the release of pro-healing growth factors. Several small studies have shown a positive effect of fat grafting in healing of wounds of a variety of aetiologies. When fat is combined with autologous platelet-rich plasma (PRP), there may be enhanced healing effects. This may be due to the pro-angiogenic and anti-inflammatory effects of PRP. We aim to synthesise the current evidence on combination fat grafting and PRP for wound healing to establish the efficacy of this technique.

**Methods/design:**

We will conduct a comprehensive literature search in the MEDLINE, EMBASE, CENTRAL, Science Citation Index, and Google Scholar databases (up to July 2017) to identify studies on fat grafting and PRP for wound healing. All primary studies and systematic reviews of these studies will be included, except case reports and case series with fewer than three patients, to evaluate the outcome of fat grafting and PRP on wound healing either on its own or when compared to other studies. Primary outcome measures are expected to be the proportion of total wounds healed at 12 weeks and the average wound healing time (time for 100% re-epithelialisation). Expected secondary outcome measures are the proportion of wounds achieving 50% wound healing, the type of wound benefitting most from fat grafting, economic evaluation, health-related quality of life, and adverse events. Subgroup analysis will be performed for the proportions of wounds healed based on wound aetiology.

**Discussion:**

This review will provide robust evidence of the efficacy of fat grafting and PRP for wound healing. This is an emerging technique, and this review is expected to guide clinical practice and ongoing research aimed at improving wound care.

**Systematic review registration:**

PROSPERO CRD42016049881

**Electronic supplementary material:**

The online version of this article (doi:10.1186/s13643-017-0505-8) contains supplementary material, which is available to authorized users.

## Background

Wound management places a significant burden on healthcare, costing the NHS in the UK billions of pounds per annum [1]. Traditional wound management options include regular dressings and skin grafting. Dressing management can often be a slow, time-consuming process whereas skin grafts are associated with high-cost, donor-site morbidity and occasionally hospital admission.

Fat grafting has become increasing popular in contouring procedures over the last few decades [2]; however, more recently, there is becoming an emphasis on its regenerative potential. Adipose-derived stem cells or MSCs found in fat are believed to facilitate healing through differentiation into cells which affect wound healing e.g. fibroblasts and keratinocytes [3]. They also release pro-healing growth factors and anti-inflammatory cytokines [4] as well as healing-related peptides such as leptin and adiponectin which together may enhance wound healing [5]. Several small studies have shown that autologous fat grafting may show significant healing qualities in chronically scarred tissue after radiotherapy [6], chronic wounds [7, 8], arterial ulcers [9], pressure ulcers [10], and diabetic foot ulcers [11]. However, the evidence remains limited with no randomised controlled trials reported.

Platelet-rich plasma (PRP) is an autologous blood-derived product enriched in platelets, growth factors, chemokines, and cytokines. It is a reservoir of essential growth factors, including platelet-derived growth factor, vascular endothelial growth factor, transforming growth factor-beta 1, and insulin-like growth factor which facilitate repair and healing. Platelet-derived biologic mediators have two primary effects on wound healing: recruiting and activating cells that affect wound healing and regulation of angiogenesis [12, 13]. Platelets may also have antimicrobial and immune modulation properties which help to reduce wound infection and facilitate healing [14]. Some studies have found enhanced healing and reduced healing time of split thickness skin grafts when used in combination with PRP [14–17] and improved healing when used as the primary treatment for chronic wounds [18–20]. However, several higher level evidence studies have shown no clear benefit for wound healing over conventional treatments [21–23].

When fat and PRP are used in combination, there may be increased survival of the fat graft which may in turn increase the healing properties of the adipose-derived stem cells. This is believed to be due to the pro-angiogenic effects of PRP which allows early vascularisation of the fat therefore reversing the early ischaemic phase of the graft [24]. Another pro-survival effect may be the release of anti-inflammatory chemokines which help reduce inflammation and swelling which encourage degeneration of the graft. Hypotheses also exist which suggest PRP may provide nutrient support to the fat cells through its plasma component and that the fibrin component allows formation of a scaffold for fat cells [25–27]. Some studies have shown that when used in combination, PRP may increase the maintenance of fat grafts for contouring procedures [28–30]. However, the evidence for combination treatment in wound healing is very limited [31] and the efficacy of the technique is yet to be established.

An extensive search will be undertaken in the MEDLINE (Ovid SP), EMBASE (OvidSP), Cochrane Central Register of Controlled Trials (CENTRAL), Science Citation Index, and Google Scholar databases to identify systematic reviews and primary studies on combination fat and PRP for wound healing. This review will provide robust evidence of the efficacy of fat grafting and PRP for wound healing. This is an emerging technique, and this review is expected to guide clinical practice and ongoing research aimed at improving wound care.

## Methods/design

### Objective

This systematic review aims to summarise the best available evidence for the technique of combination fat grafting and PRP in an effort to aid in establishing the efficacy of this technique. This will be achieved by evaluating the use of the technique in the clinical setting by measuring the proportion of wounds healed and the mean wound healing time (time to 100% re-epithelialisation).

### General methods

This protocol has been registered with the PROSPERO international prospective register of systematic reviews (registration number CRD42016049881) and will be reported adhering to the Preferred Reporting Items for Systematic Review and Meta-Analysis Protocols (PRISMA-P) 2015 statement [32]. The PRISMA-P checklist for this study is included as an additional file (Additional file 1). The final review will be reported following the PRISMA statement. In the event of no randomised controlled trial (RCT) being available to be included in the study, this review will be reported according to the Meta-Analysis of Observational Studies in Epidemiology (MOOSE) guidelines [33].

### Search strategies

We will conduct searches of the MEDLINE (Ovid SP), EMBASE (OvidSP), (CENTRAL), Science Citation Index and Google Scholar databases from 1946 up to July 2017 to identify studies of relevance to this review. Publicly available trial registers (ClinicalTrials.Gov and WHO International Clinical Trials Registry Platform) will be searched for all trials. The reference list of all articles included will be crosschecked for further articles of relevance. The search strategy will use a combination of text word and Medical Subject Headings (MeSH) terms relating to the use of epidermal graft in treating wounds. There will be no restriction by study design or outcomes as the research questions are broad. No date or publication restriction will be applied. The initial search will be conducted in English; if full articles not written in English are identified and deemed to meet the criteria for inclusion in the study, they will be translated into English. A sample search strategy for MEDLINE (OvidSP) is shown, and a similar strategy will be adapted for use in other databases.

1. ([fat graft] OR [fat transfer] OR [adipose graft] OR [adipose stem cell] OR [adipose derived stem cell] OR [adipose tissue transplantation]) AND [wound healing] AND ([platelet-rich-plasma] OR [PRP])

### Selection criteria

All human studies related to fat grafting and PRP for treating wounds will be included.

### Study design

All primary studies (excluding case reports or case series with fewer than three patients) or systematic reviews of these studies, which evaluate the outcome of fat grafting and PRP for wound healing, will be included.

### Type of participants

The participants are all patients (child and adult) with wounds of any size or aetiology treated by a combination of fat grafting and PRP.

### Setting

Studies performed in any clinical setting will be included.

### Intervention

Any technique of autologous fat harvesting which is then combined with autologous PRP will be included. Traditional techniques of fat harvesting include the Coleman technique using cannula and syringe with or without centrifuging, and the suction assisted liposuction technique. Both techniques may or may not include infiltration of tumescent solution into the donor site. Autologous PRP is harvested by obtaining a peripheral blood sample and centrifuging this using a variety of different commercially available platelet purifying devices to produce PRP. The two substances are mixed together and infiltrated directly into and around the wound bed. The amount injected varies between studies. There is no evidence on a more efficacious standardised fat and PRP technique. Information regarding the harvesting methods for both fat and PRP, the volume of each substance harvested, the volume applied, and the dressing used after grafting will be documented.

### Comparator

Studies comparing combination fat grafting and PRP to any other wound management option will be included in this review.

### Outcome measures

The primary outcome measures will be the proportion of wounds with complete healing at 12 weeks and the mean wound healing time (to complete re-epithelialisation). Secondary outcome measures will be the type of wound benefitting most from fat grafting, economic evaluation, health-related quality of life, and adverse events. Subgroup analysis will be performed for the proportions of wounds healed based on wound aetiology dependent on sufficient data sets (e.g. vascular wounds, diabetic wounds, traumatic wounds, burn wounds).

### Exclusion criteria

The exclusion criteria will be case series of less than three cases; studies describing the use of fat grafting and PRP in cases not involving wound healing, studies not reporting time or proportion for 100% wound healing, studies in animal models, and studies describing only the harvest technique without treatment outcome.

### Study selection and data management

Study selection will be conducted in a two-stage process. The titles and abstracts will initially be screened by two reviewers, using pre-specified screening criteria, for potential eligibility after excluding duplicate records. Next, studies identified as relevant will undergo full-text review by both reviewers. Any discrepancies between the reviewers will be resolved by discussion or by referral to a third reviewer. Any relevant non-English language articles will be translated where necessary. The search results, including abstracts, full-text articles, and record of the reviewer’ s decisions, including reasons for exclusion, will be recorded in Endnote X7 (Clarivate Analytics, USA).

### Data extraction

The data from all full-text articles accepted for the final analysis will be independently retrieved by two authors using a standardised data extraction form. Any disagreements and differences will be resolved by discussion or referral to a third reviewer. Primary study authors will be contacted if further information is needed or some data are missing. The following data will be extracted:

- Study characteristics (authors, year of publication, country of publication, study design)
- Patient demographics (number of subjects, gender, mean age, comorbidities, number of wounds treated)
- Wound characteristics (wound aetiology, mean wound duration, mean wound size, pre-grafting wound quality)
- Characteristics of the intervention and control group (wound bed preparation, fat grafting technique, PRP harvesting technique, volume harvested, volume applied, number of applications, anaesthesia, use of antibiotics, wound dressing post-grafting, bed rest post-grafting)
- Outcomes (mean wound healing time, proportion of wounds with 100% healing, type of wounds with 100% healing, number of failed grafts, the type of wound benefitting most from fat grafting, economic evaluation, health-related quality of life, and adverse events)
- Length of follow-up
- Cost of fat grafting and PRP combination plus dressings and follow-up
- Health-related quality of life and patient-reported outcome measures in patients managed with fat and PRP
- Complications or adverse events occurring within the study duration
- Statistical analysis model used

### Assessment of risk of bias of included studies

Included studies will be critically appraised for methodological quality and risk of bias by two review authors independently. Discrepancies will be resolved through consensus or referral to a third reviewer if necessary. The included studies are expected to all be observational studies, with no RCTs. Should there be any RCTs, they will be assessed according to the Cochrane Collaboration Risk of Bias Assessment Tool [34]. The observational cohort studies will be assessed using the Cochrane Risk of Bias Assessment Tool for Non-Randomized Studies of Interventions (ACROBAT-NRSI) [35]. This will evaluate the risk of bias due to confounding, selection, measurement, and interpretation. The quality of reporting will be assessed using the Strengthening the Reporting of Observational Studies in Epidemiology (STROBE) checklist [36].

### Data analysis and synthesis

The main outcome measure of the included studies will be the pooled estimate of the proportions of wounds healed at 12 weeks and the mean wound healing time with corresponding 95% confidence intervals. Meta-analysis will be performed should a sufficient number of studies with consistent characteristics be found in terms of study design and outcome reporting. We will explore the sources of potential clinical and methodological heterogeneity based on the study design, population, intervention, and comparator characteristics and outcomes. Statistical heterogeneity will be assessed using the chi-square test and quantified with the *I* ^2^ statistic. The thresholds for interpretation of *I* ^2^ will be in accordance with the definitions presented in the Cochrane Handbook for Systematic Reviews of Interventions [37]. Narrative synthesis will be performed in the event that the meta-analysis is not appropriate. The narrative synthesis will be grouped by the outcome of interest. As several different fat grafting and PRP harvesting and application techniques are expected to be used, the difference between the techniques will likely be synthesised narratively. We also expect wounds from various aetiologies to be treated. The wounds will be broadly classified into acute (<3 months in duration) and chronic (≥3 months in duration), and the difference in outcome will then be synthesised narratively. As the number of studies included in the review in anticipated to be small, the need for a sensitivity analysis is not expected.

Data synthesis will be performed using Review Manager 5.3 provided by The Cochrane Collaboration [37]. Should no RCT be available, a meta-analysis of observational study will be performed using StatsDirect statistical software (StatsDirect statistical software, version 2.8.0; StatsDirect, Altrincham, UK). The quality of evidence will be rated using the Grading of Recommendations Assessment, Development and Evaluation (GRADE) approach. The GRADE analysis will be performed as per Cochrane Handbook for Systematic Reviews of Interventions and applied for all outcomes. All studies will be ranked based on the level of evidence and graded based on quality of evidence by two authors independently. Data will be presented in a summary of findings table using GRADE-pro for the mean wound healing time and proportion of wounds with 100% healing.

## Discussion

This review aims to determine the efficacy of a combination treatment of fat grafting and platelet-rich plasma for wound healing. This will be done by evaluating the overall proportion of wounds completely healed with this technique and the mean wound healing time. Although there is some evidence that when used in isolation these techniques improve wound healing, the efficacy of them used in combination is currently unclear. It is important to evaluate these two emerging autologous treatments for their regenerative potential in order to guide clinical practice and future research. To our knowledge, this is the first systematic review to evaluate the efficacy of this technique for wound healing.

### Limitations

We expect the sensitivity of our search to be limited by the lack of MeSH terms for platelet-rich plasma and a combination of fat grafting and PRP. We have included a wide range of text word combinations to overcome this. We also expect the small number of studies to limit the potential for meta-analysis of studies although we will continue to proceed with a narrative review in this instance.

## Additional file

Additional file 1: PRISMA-P checklist. (DOCX 509 kb)

## Abbreviations

ACROBAT-NRSI: Cochrane Risk of Bias Assessment Tool for Non-Randomized Studies of Interventions
CENTRAL: Cochrane Central Register of Controlled Trials
EMBASE: Excerpta Medica Database
GRADE: Grading of Recommendations Assessment, Development and Evaluation
MeSH: Medical Subject Headings
MOOSE: Meta-Analysis of Observational Studies in Epidemiology
NHS: National Health Service
PRISMA-P: Preferred Reporting Items for Systematic Review and Meta-Analysis Protocols
PRP: Platelet rich plasma
RCT: Randomised controlled trial
STROBE: Strengthening the Reporting of Observational Studies in Epidemiology
UK: United Kingdom of Great Britain and Northern Ireland
