# Supplementary material for: Protocol for a systematic review of the efficacy of fat grafting and platelet-rich plasma for wound healing
Source: Syst Rev. 2017 Jun 6;6:111. doi: 10.1186/s13643-017-0505-8 (PMC5461711; doi:10.1186/s13643-017-0505-8)
Supplement: Additional file 1: — PRISMA-P checklist. (DOCX 509 kb) [file 13643_2017_505_MOESM1_ESM.docx]

**PRISMA-P 2015 Checklist**

# **This checklist has been adapted for use with systematic review protocol submissions to BioMed Central journals from Table 3 in Moher D et al:** Preferred reporting items for systematic review and meta-analysis protocols (PRISMA-P) 2015 statement. *Systematic Reviews* 2015 **4**:1

# An Editorial from the Editors-in-Chief of *Systematic Reviews* details why this checklist was adapted - **Moher D, Stewart L & Shekelle P:** Implementing PRISMA-P: recommendations for prospective authors. *Systematic Reviews* 2016 **5**:15

| Section/topic | # | Checklist item | Information reported | | Line number(s) |
| --- | --- | --- | --- | --- | --- |
|  |  |  | Yes | No |  |
| ADMINISTRATIVE INFORMATION | | | | | |
| Title | | | | | |
| Identification | 1a | Identify the report as a protocol of a systematic review | Y |  | 1 |
| Update | 1b | If the protocol is for an update of a previous systematic review, identify as such |  | N |  |
| Registration | 2 | If registered, provide the name of the registry (e.g., PROSPERO) and registration number in the Abstract | Y |  | 60 |
| Authors | | | | | |
| Contact | 3a | Provide name, institutional affiliation, and e-mail address of all protocol authors; provide physical mailing address of corresponding author | Y |  | 4-19 |
| Contributions | 3b | Describe contributions of protocol authors and identify the guarantor of the review | Y |  | 259-263 |
| Amendments | 4 | If the protocol represents an amendment of a previously completed or published protocol, identify as such and list changes; otherwise, state plan for documenting important protocol amendments |  | N |  |
| Support | | | | | |
| Sources | 5a | Indicate sources of financial or other support for the review | Y |  | 269 |
| Sponsor | 5b | Provide name for the review funder and/or sponsor |  | N/A |  |
| Role of sponsor/funder | 5c | Describe roles of funder(s), sponsor(s), and/or institution(s), if any, in developing the protocol |  | N/A |  |
| INTRODUCTION | | | | | |
| Rationale | 6 | Describe the rationale for the review in the context of what is already known | Y |  | 66-108 |
| Objectives | 7 | Provide an explicit statement of the question(s) the review will address with reference to participants, interventions, comparators, and outcomes (PICO) | Y |  | 112-115 |
| METHODS | | | | | |
| Eligibility criteria | 8 | Specify the study characteristics (e.g., PICO, study design, setting, time frame) and report characteristics (e.g., years considered, language, publication status) to be used as criteria for eligibility for the review | Y |  | 138-160, 169-172 |
| Information sources | 9 | Describe all intended information sources (e.g., electronic databases, contact with study authors, trial registers, or other grey literature sources) with planned dates of coverage | Y |  | 126-134 |
| Search strategy | 10 | Present draft of search strategy to be used for at least one electronic database, including planned limits, such that it could be repeated | Y |  | 135-6 |
| ***STUDY RECORDS*** | | | | | |
| Data management | 11a | Describe the mechanism(s) that will be used to manage records and data throughout the review | Y |  | 117-123 |
| Selection process | 11b | State the process that will be used for selecting studies (e.g., two independent reviewers) through each phase of the review (i.e., screening, eligibility, and inclusion in meta-analysis) | Y |  | 174-181 |
| Data collection process | 11c | Describe planned method of extracting data from reports (e.g., piloting forms, done independently, in duplicate), any processes for obtaining and confirming data from investigators | Y |  | 183-205 |
| Data items | 12 | List and define all variables for which data will be sought (e.g., PICO items, funding sources), any pre-planned data assumptions and simplifications | Y |  | 188-205 |
| Outcomes and prioritization | 13 | List and define all outcomes for which data will be sought, including prioritization of main and additional outcomes, with rationale | Y |  | 162-167 |
| Risk of bias in individual studies | 14 | Describe anticipated methods for assessing risk of bias of individual studies, including whether this will be done at the outcome or study level, or both; state how this information will be used in data synthesis | Y |  | 207-216 |
| ***DATA*** | | | | | |
| Synthesis | 15a | Describe criteria under which study data will be quantitatively synthesized | Y |  | 218-239 |
|  | 15b | If data are appropriate for quantitative synthesis, describe planned summary measures, methods of handling data, and methods of combining data from studies, including any planned exploration of consistency (e.g., *I* ^2^, Kendall’s tau) | Y |  | 224 |
|  | 15c | Describe any proposed additional analyses (e.g., sensitivity or subgroup analyses, meta-regression) | Y |  | 166-167 |
|  | 15d | If quantitative synthesis is not appropriate, describe the type of summary planned | Y |  | 227-230 |
| Meta-bias(es) | 16 | Specify any planned assessment of meta-bias(es) (e.g., publication bias across studies, selective reporting within studies) | Y |  | 212-215 |
| Confidence in cumulative evidence | 17 | Describe how the strength of the body of evidence will be assessed (e.g., GRADE) | Y |  | 238 |

# 
